# Molecular Mechanoneurobiology: An Emerging Angle to Explore Neural Synaptic Functions

**DOI:** 10.1155/2015/486827

**Published:** 2015-04-14

**Authors:** Wei Hu, Chenyi An, Wei J. Chen

**Affiliations:** ^1^School of Medicine, Zhejiang University, Hangzhou 310058, China; ^2^School of Mechanical Engineering, Zhejiang University, Hangzhou 310058, China; ^3^Collaborative Innovation Center for Diagnosis and Treatment of Infectious Diseases, Zhejiang University, Hangzhou 310058, China

## Abstract

Neural synapses are intercellular asymmetrical junctions that transmit biochemical and biophysical information between a neuron and a target cell. They are very tight, dynamic, and well organized by many synaptic adhesion molecules, signaling receptors, ion channels, and their associated cytoskeleton that bear forces. Mechanical forces have been an emerging factor in regulating axon guidance and growth, synapse formation and plasticity in physiological and pathological brain activity. Therefore, mechanical forces are undoubtedly exerted on those synaptic molecules and modulate their functions. Here we review current progress on how mechanical forces regulate receptor-ligand interactions, protein conformations, ion channels activation, and cytoskeleton dynamics and discuss how these regulations potentially affect synapse formation, stabilization, and plasticity.

## 1. Introduction

**Figure 1:**
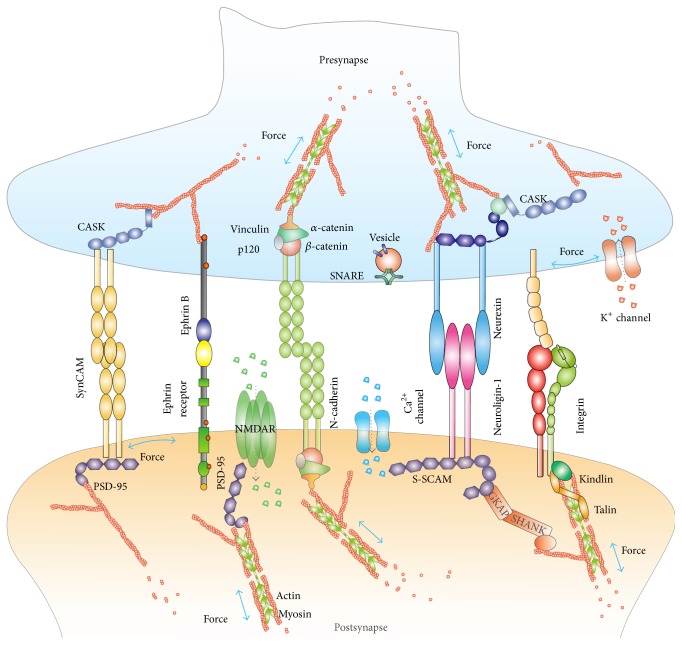
Schematic of a neural synapse with key molecules under external and/or internal mechanical forces. Neural synapses are very tight, dynamic, and well organized by many synaptic adhesions and signaling receptors (e.g., cadherins, integrins, and Eph/Ephrin), ion channels (e.g., NMDAR and L-type VGCC), and their associated cytoskeleton (e.g., actins). These molecules serve as mechanosensors and mechanotransducers. Cytoskeleton serves as a regulatory center that physically links membrane receptors and their associated cytoplasmic molecules (e.g., talin, PSD-95, S-SCAM, and catenin) for mechanotransduction. Mechanical forces, including extracellular forces from axon growth or other neural movements and internal forces from cytoskeletal dynamics and contractions of motor molecules (e.g., myosin), may regulate these proteins' conformations and functions, which may further determine synaptic formation and plasticity.

In the nervous system, synapses are intercellular asymmetrical adherent junctions in transmitting biophysical and biochemical information between neurons and target cells (Figure 1). They are tight and highly dynamic structures that rapidly respond and adapt to diverse intrinsic or extrinsic complex cues. From mechanical standpoints, the synapse formation at least involves four steps [1, 2]: the elongation of neurites, physical attachments between neuronal branches and their targets, survival of the axonal branch decided by mechanical forces, and complete synapse formation. Generally, mechanical force manifests some physical properties, such as stress, tension, stretch, and stiffness [3], which may regulate axonal initiation, neurite elongation or growth, and axonal retraction [4, 5] and may also mediate synapse formation and plasticity. The dynamic coupling of the cytoskeleton with the neuron's mechanical environment through transmembrane proteins (e.g., integrins) can exert forces on their substrates for the extension and anchorage of growth cones [1, 6, 7]. The mechanical tension, generated by the growth cones, promotes the stabilization of axon branches and regulates the topology of developing networks through cytoskeleton rearrangement, modulating subsequent formation of synapses [4, 8]. Notably, the rigidity of extracellular environment has been shown to influence the movements of neurites [9]. For example, neurite outgrowth of dorsal root ganglion (DRG) neurons was dependent on substrate rigidity [10]. Similarly, the astrocytes also respond to substrate rigidity with more complex morphology on stiffer substrates than those on more compliant substrates [11]. There is a mechanical stress threshold (~274 pN/mm^2^) to trigger a series of retraction and direction-changing events for growth cones, which may be related to mechanosensitive ion channels that convert mechanical inputs into biochemical signals [12]. Mechanical cues in the microenvironment may also modulate differentiation and development of neurons [13]. Saha et al. [14] proposed that the biochemical and mechanical cues in the microenvironment can cooperatively regulate the differentiation of adult neural stem cells. These complex cues, for instance, can modulate notch activation and signaling to influence neuronal differentiation or development [15–17]. As to notch activation, Kopan and Ilagan [18] proposed two feasible models including the mechanotransduction model (i.e., the mechanical strain may expose site 2 of a notch receptor for protease cleavage) and the allosteric model (ligand binding may induce an allosteric change into a protease-sensitive conformation). Indeed, Meloty-Kapella et al. [19] demonstrated that the mechanical force generated by the ligand-induced endocytosis, which was dependent on dynamin, epsins, and actin, changed notch receptor's conformations to trigger effective proteolysis.

Mechanical forces can also affect the physiological and pathological development of the nervous system. Franze [20] has put forward a differential expansion hypothesis: the intrinsic mechanical force produced through growth processes, such as proliferation of neurons, can fold the cortex during the cerebral development. If the mechanical properties of intracellular and extracellular environments change, folding abnormalities of the cerebral cortex give rise to diverse clinical symptoms and cognitive deficits, such as Williams syndrome [21], autism spectrum disorders [22], and schizophrenia [23]. Likely, Alzheimer's disease may also be related to abnormality of brain tissue stiffness [24]. It has been reported that the stiffness of neuronal cells increased significantly after the treatment with amyloid-*β* protein which was from proteolytic cleavage of the amyloid-*β* precursor protein by *β*- and *γ*-secretases [25]. This result may lead us to rethink the pathogenesis of Alzheimer's disease from the mechanical standpoints.

Many evidences have been accumulated to suggest that neuronal developments are closely related to the mechanical cues from neurons themselves and their microenvironments. As to the neuronal disease treatment, carefully controlled force is obviously an effective stimulator to intervene the neuronal activities. However, the molecular mechanisms by which the force regulates neural functions, especially synaptic functions, are still largely unknown. This review will mainly focus on the mechanical regulation of adhesion receptors, ion channels, and cytoskeleton in synapses. We hope this paper can refresh our fundamental understanding on the molecular basis of neural synaptic functions and inspire new ideas to explore the mysterious nervous system, especially from the mechanobiology standpoint.

## 2. Adhesion Molecules

### 2.1. Cadherins

Cadherins are a family of type-I transmembrane proteins that mediate cell-cell adhesion at intercellular adherent junctions. A cadherin contains extracellular domains that bind homopilically with another cadherin and a cytoplasmic domain that binds with the catenin family (e.g., *α*, *β*, *γ*, or p120 catenin) [26]. The cadherin family consists of several subfamilies, such as classical cadherins, protocadherins, Fat cadherins, cadherin-like neuronal receptors, and seven-pass transmembrane cadherins [27]. They are widely expressed on various cell surfaces, including endothelial cells and neurons. Multiple classic cadherins expressed on neurons [28] mainly regulate neuronal recognition and connectivity [29]. For example, N-cadherin–mediated extracellular neuron–neuron interactions are indispensable for maintaining dendrite growth and branching [30] and synapse formation or stabilization [31]. Adjusting the expression levels of N-cadherins on neurons by overexpression or knocking-down can strengthen or attenuate spine stability respectively [32]. Moreover, the N-cadherin-induced signaling cascades regulate spine morphology, postsynaptic organization, presynaptic organization, and synapse functions [33].

Recently, many evidences have suggested that cadherins are mechanosensitive in a way that they can sense external and internal mechanical forces to trigger appropriate biological functions via a positive feedback loop (Figure 1) [26]. For example, substrate rigidity can affect the formation of cadherin junctions via changing the cellular traction forces. The softer the substrate, the less the traction forces that can be generated on cadherin adherent junctions. These traction forces are mainly generated and regulated actomyosin assembly via a positive feedback loop [34]. That is, the reorganization of the actomyosin complexes generates appropriate mechanical forces to stabilize the cadherin adhesions and the recruitment of actin fibers. Thus, the cadherin adhesion complex performs as a mechanosenor and mechanomodulator by changing its adhesion strength in response to the different rigidity of the intra- and extracellular environments.

**Figure 2:**
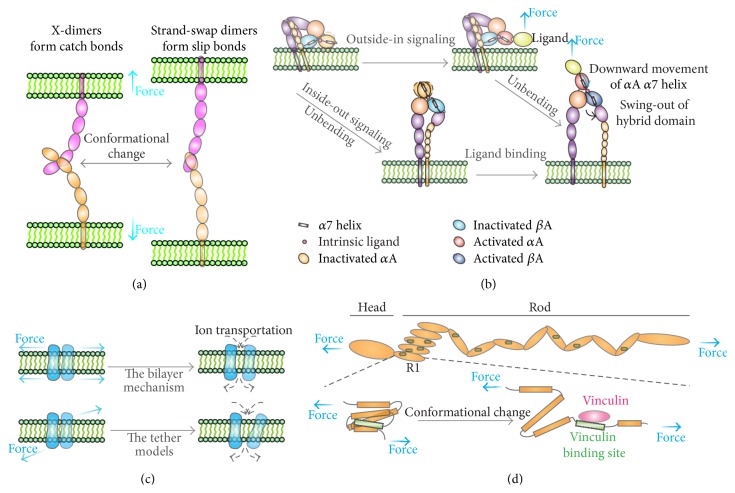
Schematics of mechanical force activation of mechanosensitive proteins. (a) Cadherins. Cadherins consist of two distinct trans-binding conformations, a strand-swap dimer (forming slip bonds, i.e., force accelerates dissociation), and an X-dimer (forming catch bonds, i.e., force impedes dissociation). Upon mechanical force application, trans-interacting cadherins switch their X-dimeric conformations to the strand-swap dimer, converting catch bonds to slip bonds. (b) Integrins. Two signaling pathways exist for integrins. In the inside-out signaling pathway, intercellular proteins (e.g., talin and/or kindlin) transduce mechanical forces across the membrane, unbending integrins and exposing ligand-binding sites. In the outside-in signaling pathway, integrin's ligand (e.g., fibronectin) binding to integrin's headpiece induces local conformational changes. Mechanical force can further activate integrin's to a long-lived state by downward moving *α*7 helix in either *α*A and/or *β*A domain, swinging out hybrid domain and separating *α* and *β* tails. Such activation can lead to recruiting the talin and/or kindlin to reorganize cytoskeleton. (c) Mechanosensitive ion channels. Mechanical force activates mechanosensitive ion channels through deforming cell membrane (i.e., the bilayer mechanism) and tethering the channels (e.g., the single-tether model and the dual-tether model). (d) Talin. Mechanical stretching of talins exposes latent binding sites for vinculins, converting the mechanical effect into biochemical signals.

Assisted by many biophysical studies, especially by structural and single-molecule studies, the molecular mechanism by which the cadherin complex senses mechanical cues has been gradually unveiled. An ultrasensitive biomembrane force probe, a state-of-the-art single-molecule biophysical technique, was used to measure rupture strengths of single-paired trans-bonded E-cadherins. In this study, the E-cadherin trans-interaction was found to exist a hierarchy of rupture strengths, suggesting multiple binding states, which are related to multiple biomechanical functions for E-cadherins [35]. Moreover, the cadherin ectodomain is a Ca^2+^-switched mechanostable structure [36]. That is, at high Ca^2+^ concentrations, the ectodomain structure is fairly rigid and stable, assuring the transmission of mechanical stimuli, while, at low Ca^2+^ concentrations, it turns into a compliant structure, which attenuates effective mechanical transmission. In addition, classical cadherins form two distinct trans-binding conformations, a strand-swap dimer, and an X-dimer [37]. Interestingly, X-dimers form catch bonds (i.e., force-prolonged bond lifetimes), strand-swap dimers form slip bonds (i.e., force-shortened bond lifetimes), and ideal bonds (i.e., force-independent bond lifetimes) appear when X-dimers change to strand-swap dimers [38] (Figure 2(a)). Later, computer simulations and single-molecule force spectroscopy were combined to study the structural mechanism of cadherin X-dimer's catch bonds [39]. Their data suggest that tensile force flexes the cadherin extracellular region of X-dimers, which induces new hydrogen bonds, resulting in a tighter contact. Recently study using optical trap-based single-molecule assay from Buckley and colleagues [40] reported that mechanical force strengthened cadherin/catenin complex binding to actin filament to resist tensile forces more efficiently via catch bonds. They also found that there were two bound states—a weakly bound state and a strongly bound state existed on cadherin/catenin complex binding to an F-actin filament. Mechanical force can switch the bound states probably through changing conformations of *α*-catenin which severs as a tension transducer [41]. That is, once mechanical force is exerted on the cadherin-catenin/F-actin bond, a weakly bound state can be switched to a strongly bound state, which stabilizes the cadherin-catenin and F-actin interactions [40]. Mechanical force can also enhance binding of the cadherin/catenin complex to vinculins by exposing vinculin-binding sites on *α*-catenins. Once vinculins are recruited to cadherin/catenin complex, actomyosins are then activated to trigger downstream molecular cascades, such as remodeling adherent interactions between cadherins. Does mechanical regulation on cadherin/catenin complex at the molecular level affect neurite growth or synapse formation in vivo? Indeed, Bard et al. [42] demonstrated that the mechanical coupling between the cadherin/*β*-catenin complex with actins on primary neurons was a major determinant of growth cone advance and neurite extension through the adhesions between neuron and N-cadherin-coated substrates.

### 2.2. Integrins

Integrins are a large family of noncovalently associated heterodimeric adhesion receptors formed by a *α*- and a *β*-chain. Their bindings with various ligands mediate cell-cell and cell-extracellular matrix (ECM) interactions and trigger signaling pathways for cell adhesion, migration, proliferation, and differentiation [43]. 18 *α* and 8 *β* subunits have been identified in mammals, forming 24 different integrin heterodimers [44]. Each integrin subunit consists of a short cytoplasmic tail, a single transmembrane domain, and a large extracellular domain. Integrin's cytoplasmic tail has been reported to interact with cytoplasmic proteins, such as talin and kindlin. These interactions physically connect integrins to actin cytoskeleton, transducing biophysical and biochemical signals bidirectionally across cell membrane [45].

In neurons, several types of integrins (e.g., *β*1 and *β*3 integrins) are expressed at synaptic membranes on both nascent and mature synapses and regulate neuronal functions [46–49]. These functions include neuronal migration, neurite growth [50] and path finding [51], dendritic spine plasticity [47], synaptic differentiation and maturation [52–54], synapse density [55], synaptic strength [56], and plasticity [57]. The integrin and its associated adaptor proteins can bind directly to some kinases (e.g., Arg kinase and focal adhesion kinase) to initiate signaling to mediate dynamic cytoskeleton organization and transcription [55, 58] and to modulate induced neuronal firing activities as well as trafficking of N-Methyl-D-aspartate (NMDA) and *α*-Amino-3-hydroxy-5-methyl-4-isoxazolepropionic acid (AMPA) receptors [47, 59]. Such regulations may lead to synaptic scaling. But the exact roles of integrin in mediating neuron transmission still remain unclear, which is worth more investigations.

Integrins and their associated proteins have been demonstrated as mechanical sensors. They not only sense mechanical cues but also convert such cues to biochemical signals and transduce them into the cell [45]. To sense mechanical cues, an integrin can switch among multiple global conformations. Generally, integrins exist at at least three states, inactive low affinity state in which an integrin adopts a compact and bent conformation with a closed headpiece [60], intermediate affinity state with extended conformations and a closed headpiece, and active and high affinity state with extended conformations and an open headpiece [61]. These conformational changes can be activated bidirectionally via the inside-out or outside-in signaling pathway [62] (Figure 2(b)). In the inside-out signaling pathway, a talin or a kindlin binds with integrin's cytoplasmic *β* tail, which transduces mechanical forces from actomyosins across the membrane, separating *α* and *β* leg, extending ectodomains, and exposing ligand-binding sites [45]. In the outside-in signaling pathway, extracellular matrix (ECM) proteins (e.g., fibronectin) binding to integrin's headpiece induces local conformational changes (i.e., *α*7 helix downward movement in either *α*A and/or *β*A domain). These conformational changes then propagate downward to swing out hybrid domain, extend ectodomains, and separate intracellular *α* and *β* tails, leading to the talin and/or kindlin recruitment and cytoskeleton reorganisation [63]. Mechanical force can facilitate extension but impede bending of cell-surface *α*_*L*_ *β*_2_ integrin when it engages with its ligand ICAM-1 (Intercellular Adhesion Molecule 1) using non-fluorescence-labeled single-molecule biomechanical assay [64]. Moreover, mechanical force can also regulate integrin's binding to its ligands. Friedland et al. [65] found that myosin II–generated cytoskeletal force regulated the bond strength between *α*_5_ *β*_1_ integrin and fibronectin by switching integrin from a relaxed state to a tensioned state. Later on, Zhu and his colleagues used single-molecule biomechanical force-clamp assays to demonstrate catch bond formed between *α*_5_ *β*_1_, *α*_*L*_ *β*_2_, and *α*_4_ *β*_1_ integrin with their respective ligands [64, 66–68]. They also found that mechanical force accelerated downward movement of *α*A domain *α*7 helix to allosterically open the MIDAS (metal ion-dependent adhesion site) on the top of *α*A domain. This allosteric regulation by force is a possible molecular mechanism for *α*A domain containing integrin catch bonds [69, 70]. Mechanical force may enforce hybrid domain swing-out, which may also play a critical role in forming integrin's catch bands [67]. Finally, the clustered complex switches to low-binding affinity state possibly through the phosphorylation of *β*_3_-integrin tails [71]. Therefore, the mechanical regulation for synaptic neurotransmissions, synapse formation, and plasticity through integrins may exist, but there is still lacking experimental evidences.

### 2.3. Eph/Ephrin

Eph receptors are known as the largest family of receptor tyrosine kinases, which consist of two subclasses, the A-subclass Eph receptors (e.g., EphA1–EphA10) and the EphB subclass receptors (e.g., EphB1–EphB6). Similarly, ephrins, as Eph ligands, also comprise two subclasses, the A-subclass ephrins (e.g., ephrinA1–ephrinA6) and the B-subclass ephrins (e.g., ephrin-B1–ephrin-B3) based on their affinities and sequence conservation [72, 73]. The most unique feature of signaling mediated by Eph/ephrin interactions is that their downstream signaling can be bidirectionally transmitted across cell membrane [74]. Moreover, Ephs and ephrins can bind each other in* trans* or in* cis *manners [75]:* trans*-interactions activate Eph/ephrin signaling, but* cis*-interactions inhibit signaling transmission. Activated Ephs usually form clusters on cell surface, which consist of different Eph members that cross activate each other [76, 77]. Within the clusters, activated Ephs can* trans*-phosphorylate kinase domains of inactivated Ephs, initiating the downstream signaling cascades [78].

In neurons, different types of Ephs and ephrins are expressed at diverse locations over the course of neural system development. For example, EphB2s are preferentially expressed in dendrites and dendritic spines [79], EphA4 mainly at the postsynaptic density [80], and Ephrin-A3 and EphA on dendritic spines [81]. As reported, Eph and ephrin can mediate neural development [82, 83], dendritic spine formation [84], morphology [85] and maturation [86, 87], axon guidance [88], synapse formation [89], synaptic specializations [90], synaptic plasticity [91, 92], and synaptogenesis [93, 94]. For example, Eph and Ephrin binding regulates NMDAR trafficking [95, 96]. The activation of EphB2 by ephrin-B1 induces association of EphB2 receptors with the NMDAR [90] and AMPARs [93] through PDZ domain containing proteins. EphB activation by ephrinB2 can also induce the phosphorylation of NMDA receptors through Src tyrosine kinases, which leads to increasing NMDA receptor-dependent influx of calcium in response to glutamate, and finally enhances NMDA receptor-dependent gene expression [96]. Furthermore, Eph/ephrin interactions can also regulate actin cytoskeleton reorganization to some extent [87, 97].

Similar to aforementioned membrane receptors, the Eph/ephrin complex can integrate and transmit biomechanical and biochemical signaling in response to mechanical cues. Recently, tensile strains or compressive forces were found to regulate the expression of ephrin-A2 and ephrin-B2 [98]. Moreover, Salaita et al. [99] explored the mechanical regulation of the ephrin/Eph signaling. They found that the mechanical restriction of ephrin-A1s through nanofabricated chromium metal lines changes the spatial organization of EphA2s and alters the cellular response to ephrin-A1, suggesting a spatial-mechanical regulation of the EphA2 signaling pathway. Plodinec and Schoenenberger [100] proposed that the mechanical force can modify downstream cellular activities through the Eph/ephrin complex, implying that the Eph/ephrin complex serves as a mechanical transducer to convert mechanical stimuli to biochemical signals. Moreover, Eph's downstream signals can remodel the actin cytoskeleton and induce actomyosin contraction [101]. Salaita and Groves [102] proposed that EphA2 transport induced by actin cytoskeleton alters the size and the distribution of Eph-ephrin clusters, which may generate mechanical forces on the Eph-ephrin complex. But how mechanical force regulates Eph/ephrin binding and downstream signaling that affect synaptic functions, still remains not clear, due to lack of direct experimental evidences.

## 3. Ion Channels

Ion channels are pore-forming membrane proteins that allow the specific ions passage, establishing a resting membrane potential and electrical excitability, and other electrical signals [103]. In general, ion channels can roughly be classified into three groups: voltage-gated channels, ligand-gated channels, and mechanosensitive channels [104]. It is known that the external or internal force is sufficient to induce conformational changes on the gating domain of an ion channel to modulate ion transportation, converting mechanical stimuli into electrical or biochemical signals [105–107]. For example, a cystic fibrosis transmembrane conductance regulator (CFTR) is robustly activated by membrane stretch, resulting in chloride transport [108]. Membrane stretch can also regulate the activity of Nav1.5 channel in a fully reversible manner, which may serve as a phasic positive feedback component in mechanotransduction [109]. Moreover, mechanical stretch increases the number of active Nav1.5 channels and peak current, slows down recovery from, and stabilizes the inactivated states [110]. And, for Nav1.5, stretch-effects can also be partially reversible [110]. By cell-attachedpatch-clamp experiments, Chubinskiy-Nadezhdin et al. [111] found that stretch-activated channels induced local changes in Ca^2+^ concentration, triggering nonmechanosensitive K^+^ currents. Stretch-activated channels that modulate the Ca^2+^ entry can regulate mechanical strength and the organization of focal adhesion sites between a fibroblast and a substratum [112]. For Shaker Channels, membrane stretch can accelerate their activations [113, 114]. What is more, mechanical force can also regulate the activity of TREK-1 potassium channel [115], the canonical transient receptor potential channel 1 [116], and Kv channel [117].

Recently, Martinac [118] summarized three force-transmission models to elucidate the mechanisms of ion channel activations (Figure 2(c)): (1) the bilayer mechanism (the activation of the ion channels by mechanical force is only through the lipid bilayer, not by an associated protein) [119], (2) the single-tether model (the conformation of the channel depends on deformation of lipid membrane caused by a cytoskeleton or matrix proteins pushing or pulling the cell membrane where the channel resides) [120], and (3) the dual-tether model (two anchoring points, such as the ECM and large-diameter microtubules, can exert mechanical force on the channels and activate them) [120] and proposed that the connection between cytoskeleton and ion channels is necessary to mediate mechanosensory and mechanotransduction. Moreover, mechanical forces are coupled to electrical signals through multiple binding partners of the adaptor proteins, posttranslational modifications (e.g., phosphorylation), and transport and assembly of channels [121]. And mechanical forces mediated by integrins can also activate ion channels [122]. Through magnetic pulling cytometry and high speed microfluorimetric calcium imaging techniques, Matthews et al. [123] demonstrated that the mechanical force mediated by *β*_1_-integrins induces ultrarapid activation of TRPV4, triggering a rapid instantaneous calcium influx (within 4 milliseconds). They proposed that intracellular cytoskeleton may provide a physical linker between integrin and ion channels that enables direct mechanotransduction.

In neurons, ion channels (e.g., L-type voltage-gated calcium channels, small conductance calcium-activated potassium channels, gamma-aminobutyric acid receptors, *α*-Amino-3-hydroxy-5-methyl-4-isoxazolepropionic acid receptors, and N-methyl-D-aspartate receptors) [124–128] expressed at both presynapse and postsynapse can modulate synapse strength and plasticity and signal propagation between neurons [129]. Undoubtedly, some mechanosensitive ion channels on neurons are also regulated by mechanical forces. For example, the mammalian neuronal potassium channel subfamily K member 4 is also opened by membrane stretch, mediating growth cone motility and neurite elongation [130]. Remarkably, more are known about mechanical effect on NMDAR. Stretch-induced injury indeed increases ionic currents and intracellular free calcium concentration through reducing the Mg^2+^ blockade of NMDAR [131]. NMDARs regulate long-term potentiation and long-term depression of excitatory synaptic transmission through calcium flux, which is related to synaptic plasticity [132]. Kazi et al. found that the coupling between ligands binding domains can impose mechanical force on the pore-lining M3 helix of NMDARs, prolonging pore opening [133]. And mechanical injury initiates specific signaling mediated by NMDA receptor which can modulate AMPA receptor desensitization [134]. Similarly, Paoletti and Ascher [135] believed that mechanical stress may be the common stimulus and mediate NMDAR-dependent signaling transduction. Moreover, subunit composition of NMDAR also can influence its mechanical responses [136]: GluN_1_/GluN_2_B NMDARs are more sensitive to mechanical force than GluN_1_/GluN_2_A NMDARs and GluN_1_/GluN_2_A/GluN_2_B triheteromeric NMDARs, and GluN_1_/GluN_2_A/GluN_2_B NMDARs show an intermediate form of mechanosensitivity. And the phosphorylation of GluN_2_B (Ser-1323) by protein kinase Cs (PKCs) dynamically regulates NMDAR GluN_2_B mechanosensitivity, altering NMDA receptor activities. In detail, postsynaptic density protein 95 (PSD-95) binding with GluN_2_B subunit modestly affects the mechanical stimulus as a mechanical clutch through regulating cytoskeletal destabilization [136]. Therefore, mechanical force may regulate the synaptic formation and plasticity through ion channels.

## 4. Cytoskeleton

In a neuron, the synapse is a highly dynamic structure that rapidly responds and adapts to different intrinsic or extrinsic cues through cytoskeleton, which includes actins, microtubules, and their associated proteins. Actin filaments are enriched in both pre- and postsynaptic terminals, controlling dynamic synaptogenesis, regulating bidirectional morphological spine plasticity, and adjusting synaptic activity [137–142]. For example, during the recruitment of synaptic vesicles from the reserve pool to the readily releasable pool, actin filaments provide cytoskeletal tracks to help actin-based molecular motors (e.g., myosin) to transport the vesicles [137]. Moreover, during the synaptic vesicle exocytosis, actins have been found to negatively regulate the neurotransmitter release. Actin polymerization and synaptic actins likely contribute to endocytosis, which is critical to learning and memory. So dynamic rearrangement in actins plays a central role in synapse remodeling and functions [137].

An actin filament is assembled by numerous actin monomers via noncovalent interactions. It undergoes dynamic and controlled polymerization and depolymerization to accomplish appropriate organizations to adapt to mechanical stresses [143]. Previous studies have demonstrated that external forces distorted the filament structure [144], resulting in the assembly, stabilization, and reorganization of the actin stress fiber and the focal adhesion (FA). Interestingly, when bearing forces, actin filaments can survive from being severed by cofilin and function as tension sensors [145]. Catch bonds in G-actin/G-actin and G-actin/F-actin may provide a mechanoregulatory molecular mechanism by which mechanical forces regulate the depolymerization kinetics of force-bearing actin filaments throughout the actin filament and further control cell functions [146]. Furthermore, tension is also crucial to actin bundle formation [147]. Actin-associated proteins, such as formin, can sense mechanical forces to mediate actin polymerization [148, 149], which further regulates the traction force at FAs during cell migration through the speed of F-actin retrograde flow [150]. Retrograde flow of actin filaments is also associated with force generation in the growth cone of a neuron [151]. Like actins, microtubules are also important in regulating the neuron activities and maintaining the cellular structure. In the nervous system, forces generated by microtubule dynamics are crucial to the axons guidance and lengthening. Paul Letourneau pointed out that microtubules generate push forces to mediate the axonal elongation [152].

In addition to actins and microtubules, cytoskeleton-associated proteins also serve as critical components in mechanotransduction pathways in living cells. For example, talins physically link the actin cytoskeleton to membrane receptors (e.g., integrins) within cell-cell or cell-ECM adhesion junctions. A number of studies have focused on the mechanical regulation of talins. Polymerization and contraction of the actomyosin lead to stretching of talin [153], exposing latent binding sites for vinculins [154, 155] (Figure 2(d)), which is crucial for anchoring the actin cytoskeleton to focal adhesions [156]. Moreover, if the mechanical force is removed, vinculin's binding stabilizes talin's unfolded conformations and prevents talin's refolding [157]. Margadant et al. [158] later found that repeated stretch-relaxation on talins transduced mechanical signals by binding and releasing vinculins, suggesting stick-slip mechanism for talin-mediated mechanotransduction. Grashoff et al. [159] were able to directly measure mechanical forces across vinculins on living cells with a smartly designed FRET- (Forster Resonance Energy Transfer-) based force biosensor and found that high tension across vinculins contributed to dynamic assembly and enlargement of FA complex, while low tension induced the instability of the FA complex. Mechanical force generated by actin polymerization and actomyosin contraction enhances talins and integrin activities by conformational changes. Such enhancements lead to accumulation of the integrin/talin/vinculin complex and actin cytoskeleton in the adhesion sites. Talins were found to be present at neuronal synapses and to interact with PIP kinases in presynaptic compartments, which may suggest that talins coordinate actin dynamics and endocytosis [160]. Talin was also proposed to modulate filopodial motility and may couple both extension and retraction to actin dynamics in the neuronal growth cone while vinculin influences the structural integrity of filopodia [161].

Myosins, another large family of cytoskeleton-associated proteins, play key roles in driving the dynamics of actin filaments to organize the synaptic structures and to regulate synaptic functions. They were indicated to determine dendritic spine morphology [162], to maintain synaptic plasticity in the postsynaptic terminal [163], and to regulate neurotransmitter release [164]. From the mechanical standpoint, myosins can convert chemical energy by ATP hydrolysis into mechanical energy to power intracellular transportation or mechanical tethers. In detail, myosins can transport cargoes inside the cell on the tracks of actin filaments [165] and walk like motor proteins to generate force and displacement along actin filaments. Moreover, mechanosensitive myosin II can recruit itself into the fusogenic synapse to increase cortical membrane tension and boost fusion pore formation [166]. Takemoto et al. put forward that different kinds of mechanical force induce different effects on signaling transduction of the myosin [167]. That is, stretching and compressive stress induces phosphorylation and dephosphorylation, respectively, for myosin regulatory light chain. It has been found that force, which is produced by myosins and applied onto the complex actin network, regulates actin cytoskeleton dynamics and synaptic cargo transport [168] and results in the axonal retraction [169]. Moreover, postsynaptic myosin II applies forces onto the cytoskeleton in the spine, maintaining long-term potentiation (LTP) and stabilizing the synaptic plasticity [163]. Therefore, we believe that myosin-associated force is crucial to some signaling pathways that are closely relevant to cytoskeletal dynamics and synaptic plasticity.

## 5. Concluding Remarks

Growing evidences clearly demonstrate that neurons can correctly respond to their complex mechanical environments by sensing mechanical stimuli and can integrate these mechanical cues with biological signals to initiate and transduce biomechanical and biochemical signals towards the inside of the neurons to appropriately modulate diverse neural functions. Future investigations of these mechanoregulations of neural functions will lead us to a new era of unveiling the molecular basis of axon guidance, neurite growth, synaptogenesis, and synaptic plasticity [4, 5]. With rapid and significant advances of modern biomechanical technologies, especially single-molecule biomechanical methodologies, the era of the amalgamation of neuroscience with mechanobiology is coming. Such multidisciplinary researches will provide us greater insights of fundamental molecular machinery for better understanding the physiology of neural system and the pathology of dysfunctional neural diseases [170], such as Williams syndrome [21], autism spectrum disorders [22], schizophrenia [23], and Alzheimer's disease [24], from mechanical standpoints.

## References

[1] Franze K., Guck J. (2010). The biophysics of neuronal growth. *Reports on Progress in Physics*.

[2] Ayali A. (2010). The function of mechanical tension in neuronal and network development. *Integrative Biology*.

[3] Humphrey J. D., Dufresne E. R., Schwartz M. A. (2014). Mechanotransduction and extracellular matrix homeostasis. *Nature Reviews Molecular Cell Biology*.

[4] Anava S., Greenbaum A., Jacob E. B., Hanein Y., Ayali A. (2009). The regulative role of neurite mechanical tension in network development. *Biophysical Journal*.

[5] Heidemann S. R., Buxbaum R. E. (1994). Mechanical tension as a regulator of axonal development. *NeuroToxicology*.

[6] Moore S. W., Roca-Cusachs P., Sheetz M. P. (2010). Stretchy proteins on stretchy substrates: the important elements of integrin-mediated rigidity sensing. *Developmental Cell*.

[7] Kostic A., Sap J., Sheetz M. P. (2007). RPTP*α* is required for rigidity-dependent inhibition of extension and differentiation of hippocampal neurons. *Journal of Cell Science*.

[8] O'Toole M., Lamoureux P., Miller K. E. (2008). A physical model of axonal elongation: force, viscosity, and adhesions govern the mode of outgrowth. *Biophysical Journal*.

[9] Moore S. W., Sheetz M. P. (2011). Biophysics of substrate interaction: influence on neural motility, differentiation, and repair. *Developmental Neurobiology*.

[10] Koch D., Rosoff W. J., Jiang J., Geller H. M., Urbach J. S. (2012). Strength in the periphery: growth cone biomechanics and substrate rigidity response in peripheral and central nervous system neurons. *Biophysical Journal*.

[11] Moshayedi P., Da F Costa L., Christ A. (2010). Mechanosensitivity of astrocytes on optimized polyacrylamide gels analyzed by quantitative morphometry. *Journal of Physics Condensed Matter*.

[12] Franze K., Gerdelmann J., Weick M. (2009). Neurite branch retraction is caused by a threshold-dependent mechanical impact. *Biophysical Journal*.

[13] Seidlits S. K., Khaing Z. Z., Petersen R. R. (2010). The effects of hyaluronic acid hydrogels with tunable mechanical properties on neural progenitor cell differentiation. *Biomaterials*.

[14] Saha K., Keung A. J., Irwin E. F. (2008). Substrate modulus directs neural stem cell behavior. *Biophysical Journal*.

[15] Kageyama R., Ohtsuka T. (1999). The Notch-Hes pathway in mammalian neural development. *Cell Research*.

[16] Lai E. C. (2004). Notch signaling: control of cell communication and cell fate. *Development*.

[17] Gaiano N., Fishell G. (2002). The role of Notch in promoting glial and neural stem cell fates. *Annual Review of Neuroscience*.

[18] Kopan R., Ilagan M. X. G. (2009). The canonical notch signaling pathway: unfolding the activation mechanism. *Cell*.

[19] Meloty-Kapella L., Shergill B., Kuon J., Botvinick E., Weinmaster G. (2012). Notch ligand endocytosis generates mechanical pulling force dependent on dynamin, epsins, and actin. *Developmental Cell*.

[20] Franze K. (2013). The mechanical control of nervous system development. *Development*.

[21] Van Essen D. C., Dierker D., Snyder A. Z., Raichle M. E., Reiss A. L., Korenberg J. (2006). Symmetry of cortical folding abnormalities in Williams syndrome revealed by surface-based analyses. *Journal of Neuroscience*.

[22] Nordahl C. W., Dierker D., Mostafavi I. (2007). Cortical folding abnormalities in autism revealed by surface-based morphometry. *Journal of Neuroscience*.

[23] White T., Hilgetag C. C. (2011). Gyrification and neural connectivity in schizophrenia. *Development and Psychopathology*.

[24] Murphy M. C., Huston J., Jack C. R. (2011). Decreased brain stiffness in Alzheimer's disease determined by magnetic resonance elastography. *Journal of Magnetic Resonance Imaging*.

[25] Lulevich V., Zimmer C. C., Hong H.-S., Jin L.-W., Liu G.-Y. (2010). Single-cell mechanics provides a sensitive and quantitative means for probing amyloid-*β* peptide and neuronal cell interactions. *Proceedings of the National Academy of Sciences of the United States of America*.

[26] Leckband D. E., de Rooij J. (2014). Cadherin adhesion and mechanotransduction. *Annual Review of Cell and Developmental Biology*.

[27] Yagi T., Takeichi M. (2000). Cadherin superfamily genes: functions, genomic organization, and neurologic diversity. *Genes & Development*.

[28] Bekirov I. H., Needleman L. A., Zhang W., Benson D. L. (2002). Identification and localization of multiple classic cadherins in developing rat limbic system. *Neuroscience*.

[29] Takeichi M. (2007). The cadherin superfamily in neuronal connections and interactions. *Nature Reviews Neuroscience*.

[30] Tan Z.-J., Peng Y., Song H.-L., Zheng J.-J., Yu X. (2010). N-cadherin-dependent neuron-neuron interaction is required for the maintenance of activity-induced dendrite growth. *Proceedings of the National Academy of Sciences of the United States of America*.

[31] Arikkath J. (2010). *N*-cadherin: stabilizing synapses. *Journal of Cell Biology*.

[32] Mendez P., De Roo M., Poglia L., Klauser P., Muller D. (2010). N-cadherin mediates plasticity-induced long-term spine stabilization. *Journal of Cell Biology*.

[33] Arikkath J., Reichardt L. F. (2008). Cadherins and catenins at synapses: roles in synaptogenesis and synaptic plasticity. *Trends in Neurosciences*.

[34] Ladoux B., Anon E., Lambert M. (2010). Strength dependence of cadherin-mediated adhesions. *Biophysical Journal*.

[35] Perret E., Leung A., Feracci H., Evans E. (2004). Trans-bonded pairs of E-cadherin exhibit a remarkable hierarchy of mechanical strengths. *Proceedings of the National Academy of Sciences of the United States of America*.

[36] Oroz J., Valbuena A., Vera A. M., Mendieta J., Gómez-Puertas P., Carrión-Vázquez M. (2011). Nanomechanics of the cadherin ectodomain: ‘Canalization’ by Ca^2+^ binding results in a new mechanical element. *The Journal of Biological Chemistry*.

[37] Harrison O. J., Bahna F., Katsamba P. S. (2010). Two-step adhesive binding by classical cadherins. *Nature Structural & Molecular Biology*.

[38] Rakshit S., Zhang Y., Manibog K., Shafraz O., Sivasankar S. (2012). Ideal, catch, and slip bonds in cadherin adhesion. *Proceedings of the National Academy of Sciences of the United States of America*.

[39] Manibog K., Li H., Rakshit S., Sivasankar S. (2014). Resolving the molecular mechanism of cadherin catch bond formation. *Nature Communications*.

[40] Buckley C. D., Tan J., Anderson K. L. (2014). The minimal cadherin-catenin complex binds to actin filaments under force. *Science*.

[41] Yonemura S., Wada Y., Watanabe T., Nagafuchi A., Shibata M. (2010). *α*-catenin as a tension transducer that induces adherens junction development. *Nature Cell Biology*.

[42] Bard L., Boscher C., Lambert M., Mège R.-M., Choquet D., Thoumine O. (2008). A molecular clutch between the actin flow and N-cadherin adhesions drives growth cone migration. *The Journal of Neuroscience*.

[43] Campbell I. D., Humphries M. J. (2011). Integrin structure, activation, and interactions. *Cold Spring Harbor Perspectives in Biology*.

[44] Luo B.-H., Carman C. V., Springer T. A. (2007). Structural basis of integrin regulation and signaling. *Annual Review of Immunology*.

[45] Calderwood D. A., Campbell I. D., Critchley D. R. (2013). Talins and kindlins: partners in integrin-mediated adhesion. *Nature Reviews Molecular Cell Biology*.

[46] Kawaguchi S.-Y., Hirano T. (2006). Integrin *α*3*β*1 suppresses long-term potentiation at inhibitory synapses on the cerebellar Purkinje neuron. *Molecular and Cellular Neuroscience*.

[47] Shi Y., Ethell I. M. (2006). Integrins control dendritic spine plasticity in hippocampal neurons through NMDA receptor and Ca^2+^/calmodulin-dependent protein kinase II-mediated actin reorganization.. *Journal of Neuroscience*.

[48] Ning L., Tian L., Smirnov S. (2013). Interactions between ICAM-5 and *β*1 integrins regulate neuronal synapse formation. *Journal of Cell Science*.

[49] Yang X., Hou D., Jiang W., Zhang C. (2014). Intercellular protein-protein interactions at synapses. *Protein & Cell*.

[50] Rehberg K., Kliche S., Madencioglu D. A. (2014). The serine/threonine kinase Ndr2 controls integrin trafficking and integrin-dependent neurite growth. *Journal of Neuroscience*.

[51] Myers J. P., Santiago-Medina M., Gomez T. M. (2011). Regulation of axonal outgrowth and pathfinding by integrin-ECM interactions. *Developmental Neurobiology*.

[52] Carneiro A. M. D. (2010). The emerging role of integrins in neuropsychiatric disorders. *Neuropsychopharmacology*.

[53] Gupton S. L., Gertler F. B. (2010). Integrin signaling switches the cytoskeletal and exocytic machinery that drives neuritogenesis. *Developmental Cell*.

[54] Chavis P., Westbrook G. (2001). Integrins mediate functional pre- and postsynaptic maturation at a hippocampal synapse. *Nature*.

[55] Sloan Warren M., Bradley W. D., Gourley S. L. (2012). Integrin *β*1 signals through Arg to regulate postnatal dendritic arborization, synapse density, and behavior. *Journal of Neuroscience*.

[56] Pozo K., Cingolani L. A., Bassani S., Laurent F., Passafaro M., Goda Y. (2012). beta3 integrin interacts directly with GluA2 AMPA receptor subunit and regulates AMPA receptor expression in hippocampal neurons. *Proceedings of the National Academy of Sciences of the United States of America*.

[57] Thalhammer A., Cingolani L. A. (2014). Cell adhesion and homeostatic synaptic plasticity. *Neuropharmacology*.

[58] Hood J. D., Cheresh D. A. (2002). Role of integrins in cell invasion and migration. *Nature Reviews Cancer*.

[59] Juhász G., Vass G., Bozsó Z., Budai D., Penke B., Szegedi V. (2008). Integrin activation modulates NMDA and AMPA receptor function of CA1 cells in a dose-related fashion *in vivo*. *Brain Research*.

[60] Shattil S. J., Kim C., Ginsberg M. H. (2010). The final steps of integrin activation: the end game. *Nature Reviews Molecular Cell Biology*.

[61] Springer T. A., Dustin M. L. (2012). Integrin inside-out signaling and the immunological synapse. *Current Opinion in Cell Biology*.

[62] Kim M., Carman C. V., Springer T. A. (2003). Bidirectional transmembrane signaling by cytoplasmic domain separation in integrins. *Science*.

[63] Hynes R. O. (2002). Integrins: bidirectional, allosteric signaling machines. *Cell*.

[64] Chen W., Lou J., Zhu C. (2010). Forcing switch from short- to intermediate- and long-lived states of the *α*A domain generates LFA-1/ICAM-1 catch bonds. *The Journal of Biological Chemistry*.

[65] Friedland J. C., Lee M. H., Boettiger D. (2009). Mechanically activated integrin switch controls *α*
_5_
*β*
_1_ function. *Science*.

[66] Chen W., Lou J., Evans E. A., Zhu C. (2012). Observing force-regulated conformational changes and ligand dissociation from a single integrin on cells. *Journal of Cell Biology*.

[67] Kong F., García A. J., Mould A. P., Humphries M. J., Zhu C. (2009). Demonstration of catch bonds between an integrin and its ligand. *The Journal of Cell Biology*.

[68] Choi Y. I., Duke-Cohan J. S., Chen W. (2014). Dynamic control of *β*1 integrin adhesion by the plexinD1-sema3E axis. *Proceedings of the National Academy of Sciences of the United States of America*.

[69] McEver R. P., Zhu C. (2007). A catch to integrin activation. *Nature Immunology*.

[70] Lou J., Zhu C. (2007). A structure-based sliding-rebinding mechanism for catch bonds. *Biophysical Journal*.

[71] Puklin-Faucher E., Sheetz M. P. (2009). The mechanical integrin cycle. *Journal of Cell Science*.

[72] Gale N. W., Holland S. J., Valenzuela D. M. (1996). Eph receptors and ligands comprise two major specificity subclasses and are reciprocally compartmentalized during embryogenesis. *Neuron*.

[73] Pasquale E. B. (2005). Eph receptor signalling casts a wide net on cell behaviour. *Nature Reviews Molecular Cell Biology*.

[74] Holland S. J., Gale N. W., Mbamalu G., Yancopoulos G. D., Henkemeyer M., Pawson T. (1996). Bidirectional signalling through the EPH-family receptor Nuk and its transmembrane ligands. *Nature*.

[75] Arvanitis D., Davy A. (2008). Eph/ephrin signaling: networks. *Genes & Development*.

[76] Freywald A., Sharfe N., Roifman C. M. (2002). The kinase-null EphB6 receptor undergoes transphosphorylation in a complex with EphB1. *Journal of Biological Chemistry*.

[77] Janes P. W., Griesshaber B., Atapattu L. (2011). Eph receptor function is modulated by heterooligomerization of A and B type Eph receptors. *The Journal of Cell Biology*.

[78] Himanen J.-P., Nikolov D. B. (2003). Eph signaling: a structural view. *Trends in Neurosciences*.

[79] Bouvier D., Corera A. T., Tremblay M.-È. (2008). Pre-synaptic and post-synaptic localization of EphA4 and EphB2 in adult mouse forebrain. *Journal of Neurochemistry*.

[80] Tremblay M.-È., Riad M., Chierzi S., Murai K. K., Pasquale E. B., Doucet G. (2009). Developmental course of EphA4 cellular and subcellular localization in the postnatal rat hippocampus. *Journal of Comparative Neurology*.

[81] Murai K. K., Nguyen L. N., Irie F., Yu Y., Pasquale E. B. (2003). Control of hippocampal dendritic spine morphology through ephrin-A3/EphA4 signaling. *Nature Neuroscience*.

[82] Flanagan J. G., Vanderhaeghen P. (1998). The ephrins and Eph receptors in neural development. *Annual Review of Neuroscience*.

[83] Wilkinson D. G. (2001). Multiple roles of Eph receptors and ephrins in neural development. *Nature Reviews Neuroscience*.

[84] Ethell I. M., Irie F., Kalo M. S., Couchman J. R., Pasquale E. B., Yamaguchi Y. (2001). EphB/syndecan-2 signaling in dendritic spine morphogenesis. *Neuron*.

[85] Murai K. K., Pasquale E. B. (2003). 'Eph'ective signaling: forward, reverse and crosstalk. *Journal of Cell Science*.

[86] Ethell I. M., Yamaguchi Y. (1999). Cell surface heparan sulfate proteoglycan syndecan-2 induces the maturation of dendritic spines in rat hippocampal neurons. *The Journal of Cell Biology*.

[87] Shi Y., Pontrello C. G., DeFea K. A., Reichardt L. F., Ethell I. M. (2009). Focal adhesion kinase acts downstream of EphB receptors to maintain mature dendritic spines by regulating cofilin activity. *Journal of Neuroscience*.

[88] Lackmann M., Boyd A. W. (2008). Eph, a protein family coming of age: more confusion, insight, or complexity?. *Science Signaling*.

[89] Yumoto N., Wakatsuki S., Kurisaki T. (2008). Meltrin *β*/ADAM19 interacting with EphA4 in developing neural cells participates in formation of the neuromuscular junction. *PLoS ONE*.

[90] Dalva M. B., Takasu M. A., Lin M. Z. (2000). EphB receptors interact with NMDA receptors and regulate excitatory synapse formation. *Cell*.

[91] Ethell I. M., Pasquale E. B. (2005). Molecular mechanisms of dendritic spine development and remodeling. *Progress in Neurobiology*.

[92] Frank C. A., Pielage J., Davis G. W. (2009). A presynaptic homeostatic signaling system composed of the Eph receptor, ephexin, Cdc42, and Ca_V_2.1 calcium channels. *Neuron*.

[93] Kayser M. S., McClelland A. C., Hughes E. G., Dalva M. B. (2006). Intracellular and trans-synaptic regulation of glutamatergic synaptogenesis by EphB receptors. *The Journal of Neuroscience*.

[94] Akaneya Y., Sohya K., Kitamura A. (2010). Ephrin-A5 and EphA5 interaction induces synaptogenesis during early hippocampal development. *PLoS ONE*.

[95] Nolt M. J., Lin Y., Hruska M. (2011). EphB controls NMDA receptor function and synaptic targeting in a subunit-specific manner. *Journal of Neuroscience*.

[96] Takasu M. A., Dalva M. B., Zigmond R. E., Greenberg M. E. (2002). Modulation of NMDA receptor—dependent calcium influx and gene expression through EphB receptors. *Science*.

[97] Kayser M. S., Nolt M. J., Dalva M. B. (2008). EphB receptors couple dendritic filopodia motility to synapse formation. *Neuron*.

[98] Diercke K., Kohl A., Lux C. J., Erber R. (2011). Strain-dependent up-regulation of ephrin-B2 protein in periodontal ligament fibroblasts contributes to osteogenesis during tooth movement. *Journal of Biological Chemistry*.

[99] Salaita K., Nair P. M., Petit R. S. (2010). Restriction of receptor movement alters cellular response: physical force sensing by EphA2. *Science*.

[100] Plodinec M., Schoenenberger C.-A. (2010). Spatial organization acts on cell signaling: how physical force contributes to the development of cancer. *Breast Cancer Research*.

[101] Noren N. K., Pasquale E. B. (2004). Eph receptor-ephrin bidirectional signals that target Ras and Rho proteins. *Cellular Signalling*.

[102] Salaita K., Groves J. T. (2014). Roles of the cytoskeleton in regulating EphA2 signals. *Communicative & Integrative Biology*.

[103] Jentsch T. J., Hübner C. A., Fuhrmann J. C. (2004). Ion channels: function unravelled by dysfunction. *Nature Cell Biology*.

[104] Sachs F. (2010). Stretch-activated ion channels: what are they?. *Physiology*.

[105] Sukharev S., Sachs F. (2012). Molecular force transduction by ion channels—diversity and unifying principles. *Journal of Cell Science*.

[106] Hamill O. P., Martinac B. (2001). Molecular basis of mechanotransduction in living cells. *Physiological Reviews*.

[107] Markin V. S., Sachs F. (2004). Thermodynamics of mechanosensitivity. *Physical Biology*.

[108] Zhang W. K., Wang D., Duan Y., Loy M. M. T., Chan H. C., Huang P. (2010). Mechanosensitive gating of CFTR. *Nature Cell Biology*.

[109] Morris C. E., Juranka P. F. (2007). Nav channel mechanosensitivity: activation and inactivation accelerate reversibly with stretch. *Biophysical Journal*.

[110] Beyder A., Rae J. L., Bernard C., Strege P. R., Sachs F., Farrugia G. (2010). Mechanosensitivity of Na_v_1.5, a voltage-sensitive sodium channel. *Journal of Physiology*.

[111] Chubinskiy-Nadezhdin V. I., Negulyaev Y. A., Morachevskaya E. A. (2014). Functional coupling of ion channels in cellular mechanotransduction. *Biochemical and Biophysical Research Communications*.

[112] Munevar S., Wang Y.-L., Dembo M. (2004). Regulation of mechanical interactions between fibroblasts and the substratum by stretch-activated Ca^2+^ entry. *Journal of Cell Science*.

[113] Laitko U., Morris C. E. (2004). Membrane tension accelerates rate-limiting voltage-dependent activation and slow inactivation steps in a Shaker channel. *Journal of General Physiology*.

[114] Tabarean I. V., Morris C. E. (2002). Membrane stretch accelerates activation and slow inactivation in Shaker channels with S3-S4 linker deletions. *Biophysical Journal*.

[115] Maingret F., Patel A. J., Lesage F., Lazdunski M., Honoré E. (1999). Mechano- or acid stimulation, two interactive modes of activation of the TREK-1 potassium channel. *The Journal of Biological Chemistry*.

[116] Maroto R., Raso A., Wood T. G., Kurosky A., Martinac B., Hamill O. P. (2005). TRPC1 forms the stretch-activated cation channel in vertebrate cells. *Nature Cell Biology*.

[117] Laitko U., Juranka P. F., Morris C. E. (2006). Membrane stretch slows the concerted step prior to opening in a Kv channel. *Journal of General Physiology*.

[118] Martinac B. (2014). The ion channels to cytoskeleton connection as potential mechanism of mechanosensitivity. *Biochimica et Biophysica Acta—Biomembranes*.

[119] Martinac B. (2011). Bacterial mechanosensitive channels as a paradigm for mechanosensory transduction. *Cellular Physiology and Biochemistry*.

[120] Bounoutas A., Chalfie M. (2007). Touch sensitivity in *Caenorhabditis elegans*. *Pflügers Archiv: European Journal of Physiology*.

[121] Barry J., Gu C. (2013). Coupling mechanical forces to electrical signaling: molecular motors and the intracellular transport of ion channels. *Neuroscientist*.

[122] Vogel V., Sheetz M. (2006). Local force and geometry sensing regulate cell functions. *Nature Reviews Molecular Cell Biology*.

[123] Matthews B. D., Thodeti C. K., Tytell J. D., Mammoto A., Overby D. R., Ingber D. E. (2010). Ultra-rapid activation of TRPV4 ion channels by mechanical forces applied to cell surface beta1 integrins. *Integrative Biology*.

[124] Shinnick-Gallagher P., McKernan M. G., Xie J., Zinebi F., Shinnick-Gallagher P., Pitkanen A., Shekhar A., Cahill L. (2003). L-type voltage-gated calcium channels are involved in the in vivo and in vitro expression of fear conditioning. *The Amygdala in Brain Function: Basic and Clinical Approaches*.

[125] Hammond R. S., Bond C. T., Strassmaier T. (2006). Small-conductance Ca^2+^-activated K^+^ channel type 2 (SK2) modulates hippocampal learning, memory, and synaptic plasticity. *Journal of Neuroscience*.

[126] Collinson N., Kuenzi F. M., Jarolimek W. (2002). Enhanced learning and memory and altered GABAergic synaptic transmission in mice lacking the *α*5 subunit of the GABA_A_ receptor. *Journal of Neuroscience*.

[127] Lee H.-K., Takamiya K., Han J.-S. (2003). Phosphorylation of the AMPA receptor GluR1 subunit is required for synaptic plasticity and retention of spatial memory. *Cell*.

[128] Humeau Y., Shaban H., Bissière S., Lüthi A. (2003). Presynaptic induction of heterosynaptic associative plasticity in the mammalian brain. *Nature*.

[129] Voglis G., Tavernarakis N. (2006). The role of synaptic ion channels in synaptic plasticity. *EMBO Reports*.

[130] Maingret F., Fosset M., Lesage F., Lazdunski M., Honoré E. (1999). TRAAK is a mammalian neuronal mechano-gated K+ channel. *Journal of Biological Chemistry*.

[131] Zhang L., Rzigalinski B. A., Ellis E. F., Satin L. S. (1996). Reduction of voltage-dependent Mg^2+^ blockade of NMDA current in mechanically injured neurons. *Science*.

[132] Liu L. D., Wong T. P., Pozza M. F. (2004). Role of NMDA receptor subtypes in governing the direction of hippocampal synaptic plasticity. *Science*.

[133] Kazi R., Dai J., Sweeney C., Zhou H.-X., Wollmuth L. P. (2014). Mechanical coupling maintains the fidelity of NMDA receptor-mediated currents. *Nature Neuroscience*.

[134] Goforth P. B., Ellis E. F., Satin L. S. (2004). Mechanical injury modulates AMPA receptor kinetics via an NMDA receptor-dependent pathway. *Journal of Neurotrauma*.

[135] Paoletti P., Ascher P. (1994). Mechanosensitivity of NMDA receptors in cultured mouse central neurons. *Neuron*.

[136] Singh P., Doshi S., Spaethling J. M. (2012). N-methyl-D-aspartate receptor mechanosensitivity is governed by C terminus of NR2B subunit. *The Journal of Biological Chemistry*.

[137] Dillon C., Goda Y. (2005). The actin cytoskeleton: integrating form and function at the synapse. *Annual Review of Neuroscience*.

[138] Antonova I., Arancio O., Trillat A. (2001). Rapid increase in clusters of presynaptic proteins at onset of long-lasting potentiation. *Science*.

[139] Bonhoeffer T., Yuste R. (2002). Spine motility: phenomenology, mechanisms, and function. *Neuron*.

[140] Nikonenko I., Jourdain P., Alberi S., Toni N., Muller D. (2002). Activity-induced changes of spine morphology. *Hippocampus*.

[141] Kasai H., Matsuzaki M., Noguchi J., Yasumatsu N., Nakahara H. (2003). Structure-stability-function relationships of dendritic spines. *Trends in Neurosciences*.

[142] Zhang W. D., Benson D. L. (2001). Stages of synapse development defined by dependence on F-actin. *Journal of Neuroscience*.

[143] Eyckmans J., Boudou T., Yu X., Chen C. S. (2011). A Hitchhiker's guide to mechanobiology. *Developmental Cell*.

[144] Shimozawa T., Ishiwata S. (2009). Mechanical distortion of single actin filaments induced by external force: detection by fluorescence imaging. *Biophysical Journal*.

[145] Hayakawa K., Tatsumi H., Sokabe M. (2011). Actin filaments function as a tension sensor by tension-dependent binding of cofilin to the filament. *Journal of Cell Biology*.

[146] Lee C.-Y., Lou J., Wen K.-K. (2013). Actin depolymerization under force is governed by lysine 113:glutamic acid 195-mediated catch-slip bonds. *Proceedings of the National Academy of Sciences of the United States of America*.

[147] Hirata H., Tatsumi H., Sokabe M. (2007). Dynamics of actin filaments during tension-dependent formation of actin bundles. *Biochimica et Biophysica Acta—General Subjects*.

[148] Jégou A., Carlier M.-F., Romet-Lemonne G. (2013). Formin mDia1 senses and generates mechanical forces on actin filaments. *Nature Communications*.

[149] Courtemanche N., Lee J. Y., Pollard T. D., Greene E. C. (2013). Tension modulates actin filament polymerization mediated by formin and profilin. *Proceedings of the National Academy of Sciences of the United States of America*.

[150] Gardel M. L., Sabass B., Ji L., Danuser G., Schwarz U. S., Waterman C. M. (2008). Traction stress in focal adhesions correlates biphasically with actin retrograde flow speed. *Journal of Cell Biology*.

[151] Suter D. M., Miller K. E. (2011). The emerging role of forces in axonal elongation. *Progress in Neurobiology*.

[152] Letourneau P. C., Shattuck T. A., Ressler A. H. (1987). ‘Pull’ and ‘push’ in neurite elongation: observations on the effects of different concentrations of cytochalasin B and taxol. *Cell Motility and the Cytoskeleton*.

[153] Geiger B., Spatz J. P., Bershadsky A. D. (2009). Environmental sensing through focal adhesions. *Nature Reviews Molecular Cell Biology*.

[154] del Rio A., Perez-Jimenez R., Liu R., Roca-Cusachs P., Fernandez J. M., Sheetz M. P. (2009). Stretching single talin rod molecules activates vinculin binding. *Science*.

[155] Lee S. E., Kamm R. D., Mofrad M. R. K. (2007). Force-induced activation of Talin and its possible role in focal adhesion mechanotransduction. *Journal of Biomechanics*.

[156] Hirata H., Tatsumi H., Lim C. T., Sokabe M. (2014). Force-dependent vinculin binding to talin in live cells: a crucial step in anchoring the actin cytoskeleton to focal adhesions. *The American Journal of Physiology—Cell Physiology*.

[157] Yao M., Goult B. T., Chen H., Cong P., Sheetz M. P., Yan J. (2014). Mechanical activation of vinculin binding to talin locks talin in an unfolded conformation. *Scientific Reports*.

[158] Margadant F., Chew L. L., Hu X. (2011). Mechanotransduction in vivo by repeated talin stretch-relaxation events depends upon vinculin. *PLoS Biology*.

[159] Grashoff C., Hoffman B. D., Brenner M. D. (2010). Measuring mechanical tension across vinculin reveals regulation of focal adhesion dynamics. *Nature*.

[160] Morgan J. R., Di Paolo G., Werner H. (2004). A role for talin in presynaptic function. *Journal of Cell Biology*.

[161] Sydor A. M., Su A. L., Wang F.-S., Xu A., Jay D. G. (1996). Talin and vinculin play distinct roles in filopodial motility in the neuronal growth cone. *Journal of Cell Biology*.

[162] Hodges J. L., Newell-Litwa K., Asmussen H., Vicente-Manzanares M., Horwitz A. R. (2011). Myosin IIB activity and phosphorylation status determines dendritic spine and post-synaptic density morphology. *PLoS ONE*.

[163] Rex C. S., Gavin C. F., Rubio M. D. (2010). Myosin IIb regulates actin dynamics during synaptic plasticity and memory formation. *Neuron*.

[164] Seabrooke S., Stewart B. A. (2011). Synaptic transmission and plasticity are modulated by nonmuscle myosin II at the neuromuscular junction of *Drosophila*. *Journal of Neurophysiology*.

[165] Hammer J. A., Sellers J. R. (2012). Walking to work: roles for class v myosins as cargo transporters. *Nature Reviews Molecular Cell Biology*.

[166] Kim J. H., Ren Y., Ng W. P. (2015). Mechanical tension drives cell membrane fusion. *Developmental Cell*.

[167] Takemoto K., Ishihara S., Mizutani T., Kawabata K., Haga H., Olson M. F. (2015). Compressive stress induces dephosphorylation of the myosin regulatory light chain via RhoA phosphorylation by the adenylyl cyclase/protein kinase a signaling pathway. *PLOS ONE*.

[168] Kneussel M., Wagner W. (2013). Myosin motors at neuronal synapses: drivers of membrane transport and actin dynamics. *Nature Reviews Neuroscience*.

[169] Ahmad F. J., Hughey J., Wittmann T., Hyman A., Greaser M., Baas P. W. (2000). Motor proteins regulate force interactions between microtubules and microfilaments in the axon. *Nature Cell Biology*.

[170] Tyler W. J. (2012). The mechanobiology of brain function. *Nature Reviews Neuroscience*.

